# Robustness of Rashba and Dirac Fermions against Strong Disorder

**DOI:** 10.1038/srep11285

**Published:** 2015-06-12

**Authors:** Domenico Di Sante, Paolo Barone, Evgeny Plekhanov, Sergio Ciuchi, Silvia Picozzi

**Affiliations:** 1 Consiglio Nazionale delle Ricerche (CNR-SPIN), Via Vetoio, L’Aquila, Italy; 2 Department of Physical and Chemical Sciences, University of L’Aquila, Via Vetoio 10, I-67010 L’Aquila, Italy; 3 Consiglio Nazionale delle Ricerche (CNR-ISC), Via dei Taurini, Rome, Italy

## Abstract

By addressing the interplay between substitutional disorder and spin-orbit-coupling in chalcogenide alloys, we predict a strong robustness of spectral features at the Fermi energy. Indeed, supplementing our state of the art first-principles calculations with modeling analysis, we show that the disorder self-energy is vanishingly small close to the band gap, thus *i)* allowing for bulk Rashba-like spin splitting to be observed in ferroelectric alloys by means of Angle Resolved PhotoEmission Spectroscopy, and *ii)* protecting the band-character inversion related to the topological transition in recently discovered Topological Crystalline Insulators. Such a protection against strong disorder, which we demonstrate to be general for three dimensional Dirac systems, has potential and valuable implications for novel technologies, as spintronics and/or spinorbitronics.

It is nowadays well accepted that spin-orbit coupling (SOC) lies at the origin of a rich variety of appealing phenomenologies, ranging from topological insulators (TI)^1^,^2^, showing fully spin-polarized metallic surface states despite their bulk insulating character, to large bulk Rashba-like spin-splitting effects in noncentrosymmetric and polar materials^3^,^4^,^5^, characterized by strong spin-momentum locking and spin-polarized bands. In this respect, the long-known class of main-group IV-VI semiconducting chalcogenides is playing a key role. On one hand, SnTe has been proposed^6^, and later confirmed^7^, to belong to a new class of TI with unique features arising from the combination of time-reversal and crystalline symmetries, hence named Topological Crystalline Insulators (TCIs)^8^,^9^. On the other hand, GeTe—one of the few known ferroelectric semiconductors—has been put forward as the first example of so-called Ferroelectric Rashba Semiconductors (FERSCs) with large Rashba splitting^10^, where the possibility to permanently control its spin texture via a switchable electric polarization could introduce new functionalities in spintronic devices^5^. In principle, bulk Rashba-like effects and topological features could even be realized within the same material. As a matter of fact, SnTe itself undergoes a low-temperature ferroelectric (FE) transition under certain conditions^11^, whose potential implications on its TCI phase have been recently theoretically investigated^12^. The potential relevance of noncentrosymmetric TI with Rashba-like spin-splitting has been also proposed for acentric semiconductors BiTeI under pressure^13^ and BiTeCl^4^.

Beside GeTe and SnTe, FE transitions have been reported in several ternary or quaternary alloys of rock-salt IV-VI chalcogenides, such as Pb_1−*x*_Ge_*x*_Te, Pb_1−*x*_Sn_*x*_Te and PbS_*x*_Te_1−*x*_^14^,^15^,^16^. Similarly, TCI features have been observed in photoemission (ARPES) spectra of n-type Pb_1−*x*_Sn_*x*_Te and p-type Pb_1−*x*_Sn_*x*_Se alloys as a function of doping^17^,^18^,^19^,^20^,^21^,^22^. When addressing the relativistic properties of such alloys, due to the intrinsically disordered character of the solid-state solution, a major issue appears. While the role of disorder in 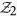 TI has been extensively investigated^23^,^24^,^25^, showing that time-reversal symmetry guarantees the presence of metallic edge states, topologically protected from non-magnetic impurities, it is not obvious how disorder entangles with spin-orbit in TCI alloys, since crystalline symmetries, crucial for topological properties, are locally broken in a random way. On the basis of general arguments, delocalized states were shown to survive at the surface of TCIs as long as the relevant symmetries, which are broken by disorder, are restored by disorder averaging, in analogy with what happens in weak 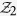 TI^26^. However, signatures of disorder should manifest in ARPES spectra^22^. Similarly, broadening/smearing effects induced by disorder could in principle screen, reduce or even prevent any bulk Rashba-type spin-splitting in FE alloys.

Based on these premises, we investigate the role of disorder in the relativistic electronic properties of IV-VI chalcogenide ternary alloys by interfacing a Coherent-Potential-Approximation (CPA) approach^27^ to highly accurate density-functional theory (DFT) relativistic calculations. We focus our attention on two prototypical ternary alloys, namely PbS_*x*_Te_1−*x*_ and Pb_1−*x*_Sn_*x*_Te. We show that the former belongs to the class of FERSC, where a large Rashba-like spin-splitting develops in disordered FE alloys. We provide a microscopic analysis of the topological nature of the TCI phase that develops in the latter. In both classes of materials we demonstrate a strong protection against broadening effects due to substitutional disorder in proximity to the band gap closure or 3D Dirac point. Remarkably, these properties ensure that both Rashba spin-split bands, as well as topological surface states (resulting from an inverted bulk orbital character screened against disorder), can be effectively detected by spectroscopic techniques such as ARPES. Most importantly, they can be used for future spintronics and/or spinorbitronics technologies based on disordered materials.

## Results

### Disordered Ferroelectric Rashba Semiconductors Systems

We start our analysis from the interplay between disorder and Rashba-type spin splittings in FE PbS_*x*_Te_1−*x*_^15^,^16^. The pristine compounds PbTe and PbS don’t display any FE instability, both crystallizing in the centrosymmetric face-centered cubic structure. On the other hand, their alloys, up to a sulfur concentration of *x*_*c*_ ~ 0.45, show FE phase transitions at doping-dependent Curie temperatures, displaying a maximum *T*_*c*_ ~ 80 *K* around *x* = 0.18^16^. By means of EXAFS, the origin of such transition has been ascribed to the off-centering of S ions in a substantially centrosymmetric PbTe host^15^, due to different size and polarizability of S and Te ions. Our DFT supercell calculations confirm this scenario, finding that below a critical concentration *x*_*c*_ ~ 0.4 sulfur displacements vary in the same range that has been experimentally observed, whereas only Te ions close to S are slightly displaced (see details in Supplementary Notes).

**Figure 1:**
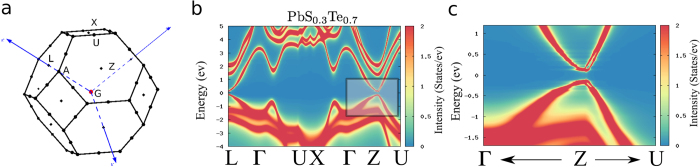
Disordered FERSC: (**a**) Distorted Rhombohedral Brillouin Zone (BZ) and high-symmetry points. (**b**) Spectral Function for PbS_0.3_Te_0.7_ FE alloy along the full irreducible BZ, where a clear spin-splitting signature appears in the black rectangle. (**c**) Zoom of the spectral function around the Z point, corresponding to the black rectangle in panel (**b**), highlighting a Rashba-like induced spin-splitting of about 50 *meV* along the ZU line perpendicular to the polar axis Γ-Z. Lorentzian broadening *δ* = 1 *meV*.

At the FE transition, the alloy structure changes from cubic to distorted rhombohedral (space group *R*3*m*), being thus isostructural with the prototypical Ferroelectric Rashba Semiconductor GeTe^5^. Rashba-like spin splittings are therefore expected to develop in the presence of SOC, especially at the valence band maximum (VBM) and conduction band minimum (CBM) around the high-symmetry point Z, at the center of hexagonal faces in the Brillouin zone (BZ) which are perpendicular to the [111] polar axis^5^ (see Fig. 1a), provided disorder-induced broadening effects do not overcome such tiny features of the spectral function. Noteworthy, this could not be the case for Tl resonant impurities in PbTe, which strongly affect the electronic bands close to the valence band edge, leading to their widening^28^. Indeed, strong signatures of random S/Te substitution appear, especially in the valence bands with predominant anionic character, as shown in Fig. 1b). Nonetheless, *the smearing of the spectral function is substantially reduced around the Fermi level*. Remarkably, we find that the VBM and CBM are more robust against disorder renormalization as the band gap decreases. In fact, the disorder self-energy appears to depend not only on the nominal S/Te concentration, but also on the size of the gap, which in turn is loosely proportional to the amount of S off-centering. Since the latter decreases as the S concentration increases above *x* ~ 0.18, spin-splitting signatures become clearly visible due to the smaller disorder self-energy (see Fig. 1c) for *x* = 0.3). On the other hand, for small S concentrations such splitting effects are substantially depressed, virtually disappearing, by disorder renormalization effects, despite the nominally weaker substitutional disorder. We stress the fact that our DFT + CPA approach is essential in capturing such disorder-induced renormalization effects. As a matter of fact, a Virtual-Crystal Approximation (VCA) approach—not taking into account the disorder self-energy—would predict large spin splittings for all concentrations corresponding to the FE phase (see details in Supplementary Notes). Being isostructural with FE GeTe and SnTe, our results suggest that novel FERSC materials, allowing for the control and manipulation of Rashba-like spin textures by tuning the FE polarization^5^,^10^, could be identified in the broad class of chalcogenide alloys. Depending on doping concentration, tiny features of ARPES spectra could become visible in PbS_*x*_Te_1−*x*_ or related PbSe_*x*_Te_1−*x*_^16^, as the self-energy induced by substitutional disorder may be significantly reduced close to the Fermi energy.

### Quasiparticle Nature of Electronic States at the TCI Phase Transition in Disordered Systems

**Figure 2:**
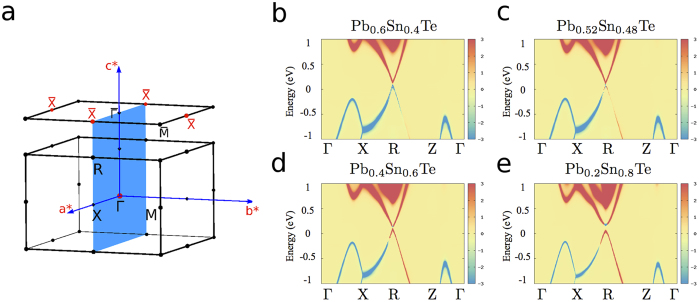
Quasiparticle Dirac electrons in disordered TCI: (**a**) Tetragonal Brillouin Zone and [001] surface. The mirror symmetry plane for the tetragonal setting which leads to the topological protection in SnTe and Pb_1−*x*_Sn_*x*_Te alloys is highlighted in blue. (**b–e**) Evolution of Pb_1−*x*_Sn_*x*_Te alloy spectral features as a function of Sn concentration for x = 0.4, 0.48, 0.6 and 0.8 along the irreducible tetragonal BZ. Red (positive) values refer to the spectral function 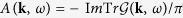 projected on the Pb_1−*x*_Sn_*x*_ cation, while blue (negative) values are projection of the spectral function on the Te anion. The R-point is the projection of the rhombohedral BZ L-point on the tetragonal BZ. Lorentzian broadening *δ* = 0.1 *meV*.

**Figure 3:**
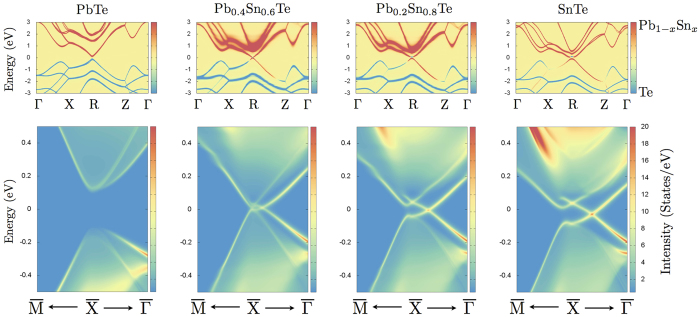
Alloying induced topological transition: Bulk spectral functions (top panels) and relative surface spectral functions (bottom panels) for Pb_x_Sn_1−*x*_Te TCI alloys (*x* = 1.0, 0.4, 0.2, 0.0). The color encoding for top panels is the same as for spectra in Fig. 2, while spectral functions at the bottom panels are given in units of states eV^−1^. The k-axis for the surface bandstructures show 1/10 of the 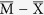 and 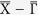 lines around 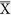 (see surface tetragonal Brillouin Zone in Fig. 2a). Lorentzian broadening used in the top panels is *δ* = 0.1 *meV*, while in the lower panels we used *δ* = 10 *meV*.

We now concentrate our attention to the topological transition occurring in Pb_1−*x*_Sn_*x*_Te alloy as a function of tin concentration. This compound, along with the Pb_1−*x*_Sn_*x*_Se analogue, has been intensively studied, both experimentally and theoretically^17^,^18^,^19^,^20^,^21^,^22^. For the first time, we propose in this Communication an ab-initio analysis of disorder effects on its spectral features by calculating the disorder self-energy in our combined DFT-CPA framework. In Fig. 2, we show the anion/cation resolved spectral functions for different dopings. The evolution of the spectral functions clearly describes the doping-induced band closure at the L points (R points in tetragonal setting), implying a change of the alloy topological character, with a critical tin concentration *x*_*c*_ ~ 0.48. As a consequence of the cation Pb/Sn substitution, disorder effects are more visible at positive energies with respect to the Fermi level, i.e. where states have a predominant cationic orbital character. The presence of disorder is reflected in the significant broadening of spectral features (spectra in wider energy windows are reported in Fig. 3), as a consequence of a larger self-energy. However, approaching the Dirac point (at the critical concentration)—where bands show a linear behavior—the spectral broadening vanishes, and a clear signature of character inversion is visible. Remarkably, such inversion appears to be protected against disorder, since the imaginary part of the disorder self-energy is found to vanish. As a result, *bands are not broadened by disorder around the bulk Dirac point*.

The trivial/topological nature of the bulk bandstructure is reflected in the lack/presence of metallic surface states through the bulk-boundary correspondance principle^2^. In particular, an inverted cation/anion character in the rock-salt chalcogenides ensures the appearance of Dirac-like surface states^6^. In fact, we find that gapless surface states appear when the tin concentration overcomes the critical value *x*_*c*_ ~ 0.48, as reported in Fig. 3, where a surface Dirac cone located close to the 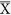 (as in pristine SnTe^6^) is clearly visible for concentrations *x* > *x*_*c*_. As it happens in the bulk band-structure, surface states appear to be well protected against disorder-induced broadenings around the Dirac point. We emphasize that surface spectral functions calculated within our approach find a direct link with experimental ARPES spectra, apart from matrix element effects (see e.g. Refs 17,19,22).

The origin of the Dirac-point protection against substitutional disorder can be further analyzed by considering the CPA description of a trivial/topological alloy in the framework of *k* ⋅ *p* model. The band structure of rock-salt chalcogenides around the L points is given by^6^,^29^:


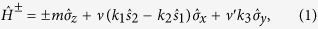


where **k** = (*k*_1_,*k*_2_,*k*_3_) forms an orthogonal system with *k*_3_ parallel to the ΓL direction and *k*_1_ along the direction perpendicular to a mirror plane. Hamiltonian (1) is represented in the basis of cation and anion p-orbitals p_*c*_/p_*a*_ (*σ*_*z*_ = ±1) and total angular momentum *j* = ±1/2 along ΓL (*s*_3_ = ±1), whereas *ν* and *v′* are materials-dependent parameters. The plus/minus sign in front of the mass term *m* refers to the trivial/topological insulator with direct/inverted band character at the gap^6^. At the critical concentration *x*_*c*_ where the bulk gap closes with bands linearly dispersing, the disorder self-energy can be written as 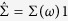, where Σ(*ω*) = −*aω* − *ibω*^2^ with *a* ~ *m*^2^/Γ^2^ and *b* ~ *m*^2^/Γ^3^, Γ being a high energy cut-off naturally introduced when dealing with linearized models (see Methods for details). The density of states assumes the simple parabolic expression *N*(*ω*) = 3*ω*^2^/2Γ^3^ coming from the tridimensionality of the Dirac cone. Remarkably, the imaginary part of the disorder self-energy, which is related to the finite life-time and bandwidth of electronic states, goes monotonically to zero when approaching the Dirac point (*ω* = 0), vanishing at the Fermi level. This is of fundamental importance for the anion/cation band-character inversion related to the topological transition^6^. Since the energy region of interest for the orbital character inversion around the L points is protected against disorder-induced broadening effects, a quasiparticle picture to electronic states can be assigned despite the nominal strong substitutional disorder. Notice that this result holds also in the presence of a small gaussian conformational disorder with variance *σ* << *m*, which is found to cause only a renormalization of coefficients *a* and *b*, where *m* is replaced with (*m*^2^ + *σ*^2^) (see details in Supplementary Notes).

## Discussion

In this work we’ve addressed the crucial role of substitutional disorder in the class of IV-VI chalcogenides, featuring SnTe and GeTe as binary prototypes of TCIs and FERSC, respectively. The main outcome of our theoretical analysis, based on the CPA model and ab-initio calculations, is the prediction that spectral features at the Fermi level are robust with respect to substitutional disorder. In closer detail, we predict a strong interplay between disorder and Rashba spin-splittings in ferroelectric ternary alloys, a key feature that has to be carefully taken into account whenever disordered systems are used in real technological applications. These FE alloys pave the way to novel spintronic concepts, opening unforeseen possibilities for the design of devices integrating logic and memory, as exemplified by the FERSC-based spin-FETs proposed in Ref. 5. Furthermore, the interplay between disorder and spin-orbit coupling in FERSC alloys gives the possibility to tune spin-orbit related features, not only by acting on the built-in polarization with an external electric field^5^, or by engineering heterostructures geometry^30^, but also by controlling the alloy’s relative concentration. Moreover, in TCI alloys we find that, as the disorder-induced self-energy vanishes at the bulk Dirac point (for the critical concentration), the orbital character inversion of quasiparticle states - “smoking gun” of the topological (crystalline) transition - is protected against the effects of disorder. Interestingly, such a protection is absent in graphene, where a non vanishing self-energy at the Fermi level leads to the so-called universal conductivity^31^. It is also worth to note here that our results for the 3D Dirac cone at the trivial/topological transition show many similarities with those obtained for Weyl electron systems and three dimensional 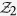 topological insulators in the weak conformational disorder limit^32^,^33^,^34^. On the other hand, it is important to highlight that finite-range potential disorder, as well as spatial correlations, beyond CPA, taken into account by Dynamical Cluster Approximation, can lead to an exponentially small density of states at the Dirac point, as a direct consequence of a small, but finite, disorder self-energy at the Fermi level^35^,^36^. As a result of the vanishing disorder self-energy at the bulk Dirac point, the spectral features of the Dirac-like conducting surface states are robust despite the nominal strong substitutional disorder. This explains why Dirac fermions are clearly detectable in experiments on chalcogenide alloys and usable for spintronic devices based on disordered topological systems.

## Methods

### DFT-CPA approach

Aiming at a quantitative analysis of the effect of disorder in the spectral features of the alloys, we combined a CPA approach^28^ with first-principles DFT relativistic calculations. CPA is valid only in the case of uncorrelated disorder, and results we obtain for the disorder induced self-energy hold only if disorder spatial correlations are neglected. As a starting point, we used DFT calculations on ordered binary compounds based on the Heyd Scuseria Ernzerhof HSE hybrid functional, as implemented in VASP^37^,^38^. Demanding hybrid-functional calculations have been shown to provide, in good agreement with GW calculations^39^,^40^, the correct band ordering at the valence band maximum (VBM) and conductance band minimum (CBM) in all lead chalcogenides, at variance with severe failures within local-density approximation. Kohn-Sham equations were solved using the projector augmented-wave method. The energy cutoff for the plane-wave expansion was 600 eV; an 8 × 8 × 8 Monkhorst-Pack k-point grid was used. For the ordered binary compounds we adopted the experimental lattice constants and ionic positions. Ab-initio relativistic HSE band structures were then projected onto a Wannier basis set^41^, and Wannier-functions tight-binding parameters were used for the CPA self-consistency loops.

In the multiorbital case the CPA can be formulated in terms of a single-site disorder-averaged propagator:


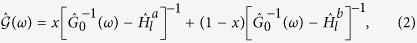


with 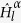 being the local (k-independent) part of the Hamiltonian describing the ordered binary compounds which are alloyed—*α* labeling SnTe or PbS (*a*) and PbTe(*b*) in this work—and 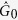 being the local propagator which embodies the average action of the environment. The disorder self-energy, defined as


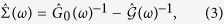


contains all informations relative to the role played by disorder, entering in the self-consistency condition 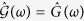 which relates the single-site propagator (2) to the local disorder-averaged propagator


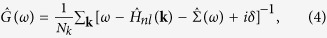


with 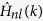 being the non-local (k-dependent) part of the Hamiltonian constructed from the pristine compounds Hamiltonians in a VCA scheme, and *δ* being a small fixed broadening added for computational and illustrative purposes.

The adopted DFT-CPA scheme provides an alternative approach to well-established techniques, such as the fully relativistic KKR-CPA^42^,^43^,^44^, often based on local-density approximation. To cite some recent example, KKR-CPA was widely and successfully used to address the effects of resonant impurities states in PbTe^17^,^45^, the effects of disorder on the quantum criticality in NbFe_2_, and chemical instabilities in Ba_1−*x*_K_*x*_Fe_2_As_2_^46^,^47^. Our approach also allows to tailor disorder effects in the surface states of alloys without resorting to computationally demanding DFT supercell calculations, at the same time improving on the conventional methodology, where tight-binding Hamiltonians are built up in slab geometry and substitutional disorder is usually taken into account at a VCA level. In fact, spectral features of the surface states are encoded in the Surface Single-site Green’s Function, which can be easily calculated from the DFT-CPA single-site disorder-averaged Green’s Function using an iterative surface renormalization method in the limit of a semi-infinite slab^48^. In this scheme, the secular equation for the Green’s function (*ω* − *H*)*G* = 1 is solved following an iterative procedure which allows to evaluate the Green’s function of the relaxed surface starting from the bulk Green’s function^48^:


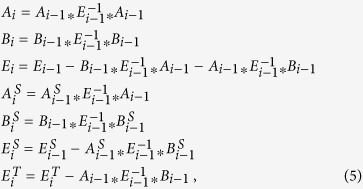



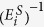
, 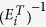 and (*E*_*i*_)^−1^ being at convergence the Green’s function of the relaxed surface, the Green’s function of the ideal surface and the Green’s function of the bulk, respectively. Our initial setup in the surface reciprocal space for the iterative procedure is


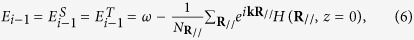


where *z* is the slab direction normal to the surface, and *H*(**R**_//_ = 0, *z* = 0) = Σ is the CPA disorder self-energy.

### CPA approach to *k* · *p* model for the Pb_1−*x*_Sn_*x*_Te alloy

Assuming a Virtual Crystal Approximation for the non-local (k-dependent) part of the chalcogenide alloys, we can write 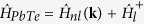 and 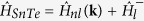, where 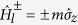 and 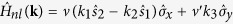. The orbital structure of the CPA local quantities is particularly simple. In fact, off diagonal components of the local propagator 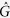 vanish, since the non-diagonal terms entering the sum of Eq. (4) are odd under the inversion **k** → −**k** when 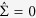. When 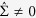, one can self-consistently prove that the self-energy acquires the following diagonal form:


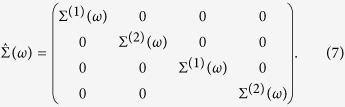


Once Eq. (7) is substituted in Eq. (3), 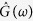 shows the same structure of 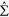, leading to the equation


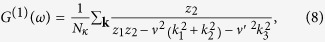


where *z*_1,2_ = *ω* − Σ^(1,2)^(*ω*). The same equation holds for *G*^(2)^(*ω*) after the exchanges 1 → 2 and 2 → 1.

Self-consistency condition can be explicitly written in terms of a simple function in the symmetric case *ν* = *ν*′ (for *ν* ≠ *ν*′, more complicated mathematical expressions involving elliptic integrals emerge, which do not modify qualitatively our conclusions). By defining the function


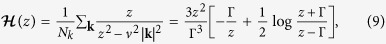


where Γ^2^ = *ν*^2^Λ^2^ is a high energy cut-off resulting from the condition 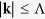, Eq. (8) can be recast as
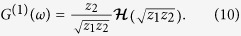


On the other hand, assuming the same (diagonal) orbital structure of 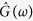 and 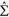, one obtains the following equations for the single-site propagator components:


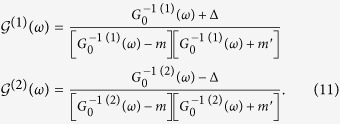


with Δ = *xm* − (1 − *x*)*m*′. The self-consistency conditions 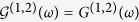 do not violate our assumption for the orbital structure of 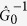 and can be solved self-consistently to get the disorder-induced self-energies.

From Eq. (11) we see that if Δ = 0 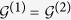. This condition defines the symmetric point *x*_*s*_ = *m*′/(*m* + *m*′) where there is no gap in the spectrum. It is worth to note that this is consistent with the VCA result, where the averaged Hamiltonian reads 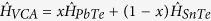 with eigenstates 
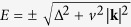
. At the symmetric point, 
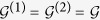
 and 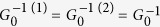, implying


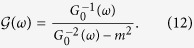


Enforcing the self-consistency condition Eq. (3), one gets Σ(*ω*) = *m*^2^*G*_0_(*ω*) and
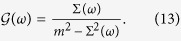


Using Eq. (10) one gets


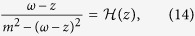


which can be solved at small energies taking Σ(*ω*) = −*aω* − *ibω*^2^. Using the definition Eq. (10) we find 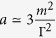 and 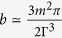 for 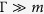. In this limit, the density of states can be evaluated as 
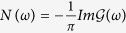
, giving the non-interacting result 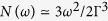. Furthermore similar robustness holds also in the case of distributed disorder (see Supplementary Notes). Having demonstrated such robustness of the spectral features at the Fermi energy against disorder in the *k* ⋅ *p* framework, we note that same results can be obtained in any materials where this three-dimensional *k* ⋅ *p* model describes the low energy features of the spectra. This could be e.g. the case of 3D topological Dirac semimetals Na_3_Bi_1−*x*_Sb_*x*_ and Cd_3_[As_1−*x*_P_*x*_]_2_^49^,^50^.

## Additional Information

**How to cite this article**: Di Sante, D. *et al.* Robustness of Rashba and Dirac Fermions against Strong Disorder. *Sci. Rep.* **5**, 11285; doi: 10.1038/srep11285 (2015).

## Supplementary Material

Supplementary Information
