# Supplementary material for: Robustness of Rashba and Dirac Fermions against Strong Disorder
Source: Sci Rep. 2015 Jun 12;5:11285. doi: 10.1038/srep11285 (PMC4650895; doi:10.1038/srep11285)
Supplement: Supplementary Information [file srep11285-s1.pdf]

Robustness of Rashba and Dirac Fermions against Strong Disorder. Supplementary Materials

Domenico Di Sante,[1,2] Paolo Barone,[1] Evgeny Plekhanov,[1] Sergio Ciuchi,[2,3] and Silvia Picozzi[1]

[1]*Consiglio Nazionale delle Ricerche (CNR-SPIN), Via Vetoio, L'Aquila, Italy*

[2]*Department of Physical and Chemical Sciences, University of L'Aquila, Via Vetoio 10, I-67010 L'Aquila, Italy*

[3]*Consiglio Nazionale delle Ricerche (CNR-ISC), Via dei Taurini, Rome, Italy*

Contents

Conformational Disorder in the CPA approach to $\boldsymbol{k}\cdot\boldsymbol{p}$ model 2

Ferroelectric instability in $PbSTe$ Alloy 4

References 6

CONFORMATIONAL DISORDER IN THE CPA APPROACH TO $\boldsymbol{k}\cdot\boldsymbol{p}$ MODEL

Let us consider a gaussian white noise added to the diagonal part fo the Hamiltonians $H_d^{\pm}$, and let us consider a simple form of the kind

$$\hat{H}_d^{+} = \begin{pmatrix} m+\xi & 0 & 0 & 0 \\ 0 & -m+\xi & 0 & 0 \\ 0 & 0 & m+\xi & 0 \\ 0 & 0 & 0 & -m+\xi \end{pmatrix}, \tag{1}$$

where ξ is a local uncorrelated term $<\xi(\mathbf{R})\xi(\mathbf{R}')>=\sigma^2\delta(\mathbf{R}-\mathbf{R}')$; a similar equation holds for the other compound. Even in the presence of conformational disorder $x_s = 1/2$ is the symmetric point for $m = m'$ where the gap vanishes. Taking into account the disorder variable ξ – which simply adds to the mass term – in the symmetric case, where $\mathcal{G}^{(1)} = \mathcal{G}^{(2)}$ and $\Delta = 0$, one gets

$$\mathcal{G}(\omega) = \left\langle \frac{G_0^{-1}(\omega)}{G_0^{-2}(\omega) - (m+\xi)^2} \right\rangle \tag{2}$$

where $< ... >$ means the average over the disorder variable ξ.

It is interesting to see whether in the symmetric point the imaginary part of the self-energy still remains zero at zero frequency. Analytically we can estimate the self-energy in the small σ limit of weak conformational disorder. In this case we expand Eq. (2) as

$$\mathcal{G}(\omega) = \frac{G_0^{-1}(\omega)}{G_0^{-2}(\omega) - m^2} \left(1 + \sigma^2 \frac{G_0^{-2}(\omega) + 3m^2}{[G_0^{-2}(\omega) - m^2]^2}\right); \tag{3}$$

then enforcing the self-consistency condition $\hat{\mathcal{G}}(\omega) = [\hat{G}_0^{-1}(\omega) - \hat{\Sigma}(\omega)]^{-1}$ one gets to first order in σ^2

$$\Sigma(\omega) = m^2 G_0(\omega) + \sigma^2 \frac{[G_0^{-1}(\omega) - m^2 G_0(\omega)][G_0^{-2}(\omega) + 3m^2]}{[G_0^{-2}(\omega) - m^2]^2} \tag{4}$$

Now it is easy to realize that if Σ as well as G are vanishing at $\omega = 0$ (the function $\mathcal{H}(z)$ vanishes as $z \longrightarrow 0$) then G_0 is also vanishing at $\omega = 0$. Thus Eq. (4) reduces to

$$\Sigma(\omega) = m^2 G_0(\omega) + \sigma^2 G_0(\omega) \tag{5}$$

which coincides with Eq. (13) reported in the main text when replacing m^2 by $m^2 + \sigma^2$ Therefore all the considerations made at the symmetric point are still valid in the presence of weak conformational disorder. In particular, the disorder self-energy acquires the same form $\Sigma(\omega) = -a\omega - ib\omega^2$, with coefficients being renormalized as $a \simeq 3\frac{m^2+\sigma^2}{\Gamma^2}$, $b \simeq 3\frac{(m^2+\sigma^2)\pi}{2\Gamma^3}$.

A separate discussion can be made in the case in which the variance of conformational disorder is large and of the order of the energy cutoff Γ. In this case a quantum phase transition between a three dimensional Dirac semimetal and a diffusive regime can occur as a function of σ as far as the imaginary part of the self-energy becomes non-zero and large at zero energy [1, 2]. This is shown in Fig. 1.

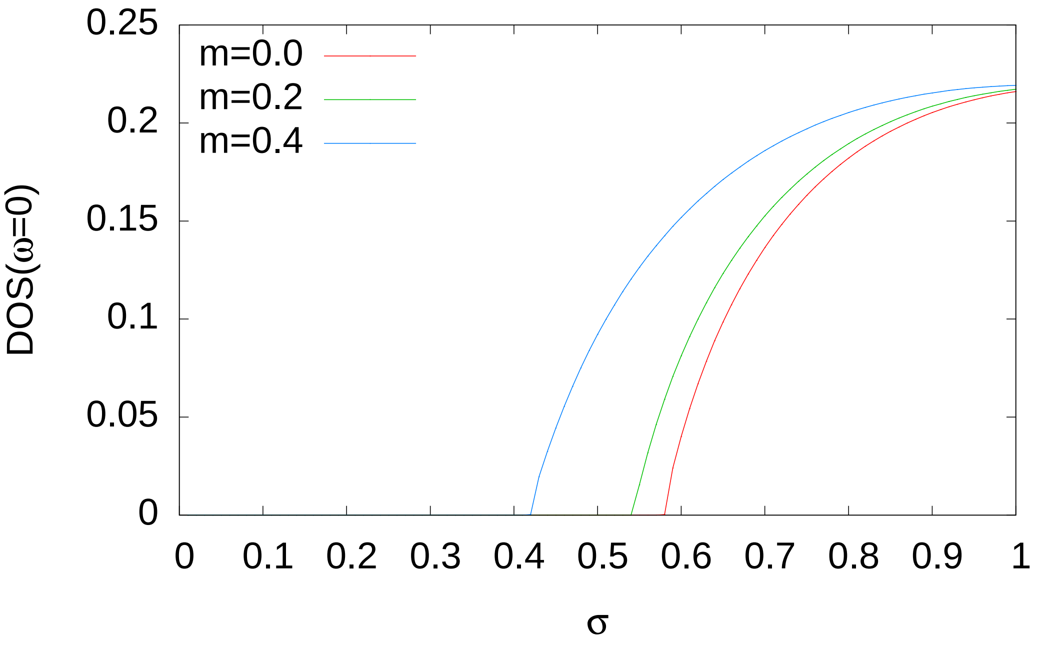

FIG. 1: The zero energy DOS at the symmetric point plotted against the disorder variance for three values of the mass.

FERROELECTRIC INSTABILITY IN *PbSTe* ALLOY

By means of supercell density functional theory (DFT) calculations, we demonstrate that even a small amount of sulfur (S) ions embedded in PbTe can induce a ferroelectric transition, which breaks the inversion symmetry. We consider two types of supercells, a 2x2x2 and a 4x4x4 PbTe unit cells (see Fig. 2a), for a total of 8 and 64 Te atoms, respectively. Substituting one or two Te ions by S ions in both supercells, we are able to study the ternary alloy PbS_xTe_{1-x} for x=0.016, 0.032, 0.125 and 0.250, ranging from a very small to high S-doped concentrations. In Fig. 2b) we report the displacements of S ions as determined from DFT calculations, compared with experimentally determined values by means of XAFS [3] for x=0.10, 0.18 and 0.30. We observe that a very small amount (x=0.016) of S ions substituted in the PbTe matrix leads the system to a ferroelectric phase transition, as

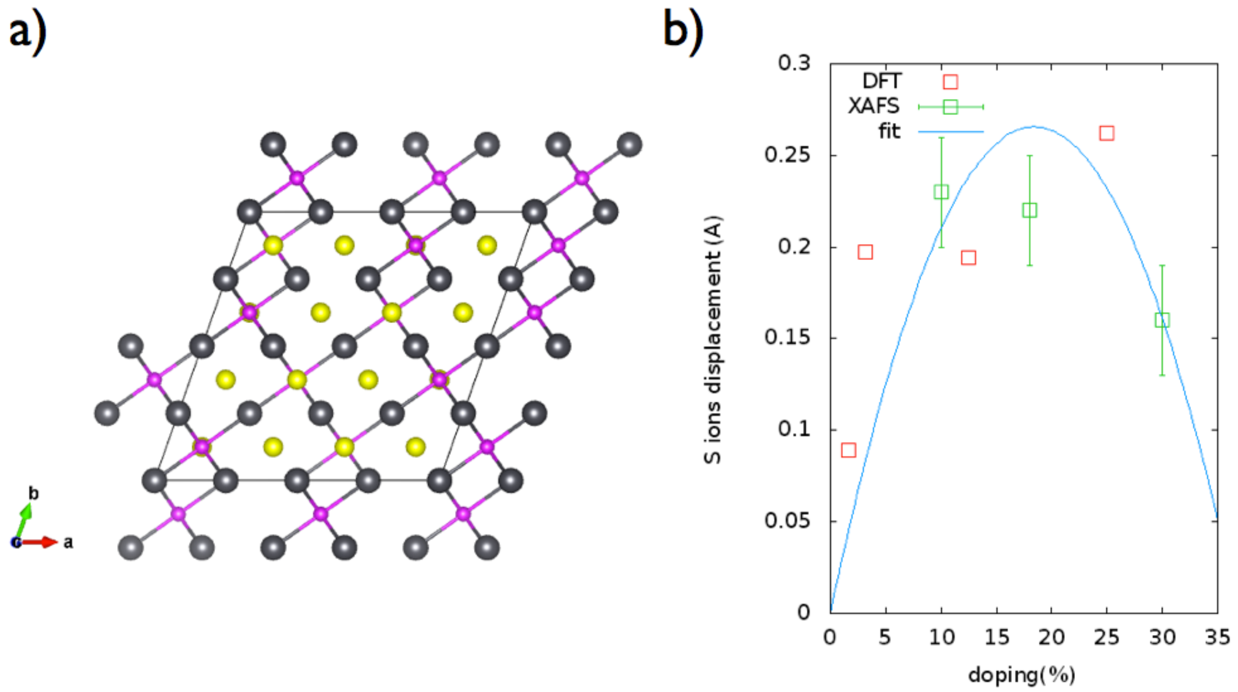

FIG. 2: a) Side view of the rhombohedral 4x4x4 (128 atoms) supercell used in the DFT calculations to study atomic relaxations at commensurate $x \sim 1.6\%$, $x \sim 3.2\%$, $x = 12.5\%$ and $x = 25\%$ concentrations. b) Displacements of S ions from the unit cell center determined from DFT supercell calculations (red squares), XAFS experiment [3] (green squares with error-bars), and best quadratic fit (blue solid line).

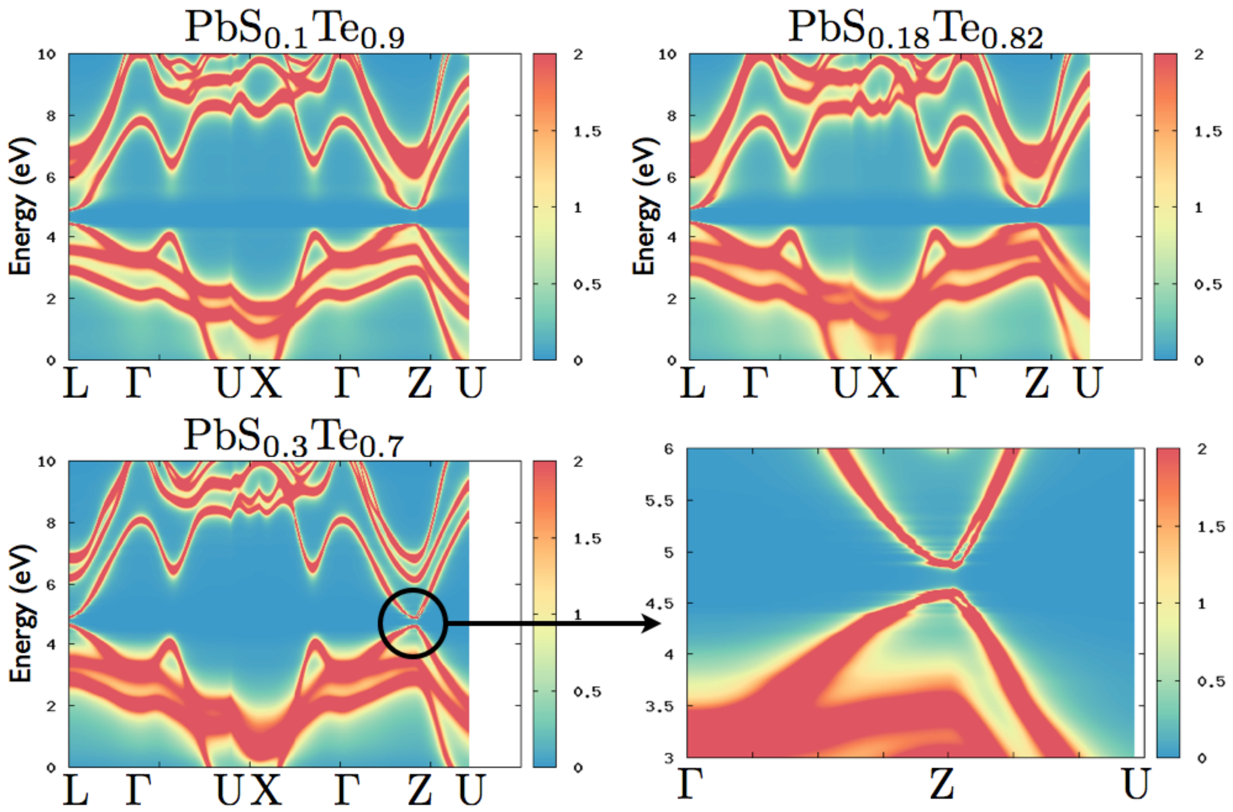

FIG. 3: CPA spectra along the rhombohedral Brillouin Zone high-symmetry lines for x=0.10, x=0.18 and x=0.30. The last panel (lower right) is a zoom around the Z point for x=0.30

experimentally predicted by an anomalous resistivity peak at the transition temperature for x=0.02 [4]. The theoretically predicted S displacements varies in the same range given by experiments (cfr the fit in FIG. 2b), and a quadratic fit over all set of data (theoretical and experimental ones) confirms that the S displacement will drop to zero around x∼0.40, a value in good agreement with the experimental x=0.45 [3]. On the other hand, our calculations confirm that Te ions remain in their centrosymmetric positions, with very small displacements only for Te atoms which are closer to S. As it happens also for Li^+ impurities in KCl [5] and $KTaO_3$ [6], the ion off-centering is favored when *i*) the difference in the ionic size (as for Te and S) causes a decrease in the repulsive force, as a consequence of the reduced ionic overlap, and *ii*) the two ions have different polarizability, which in turn reflects in a polarization force driving the impurity ion toward the neighbor ions [3]. Both conditions hold in the case of PbS_xTe_{1-x}, resulting in a cubic-to-rhombohedral distortion.

Having demonstrated the presence of a ferroelectric instability in PbS_xTe_{1-x} alloys, for

CPA calculations we used the experimental concentrations x=0.10, x=0.18 and x=0.30 with experimental bond lengths. CPA spectra for these concentrations are reported in Fig. 3. For x=0.10 and x=0.18, no Rashba splitting is visible around the Z point (see discussion in the main paper).

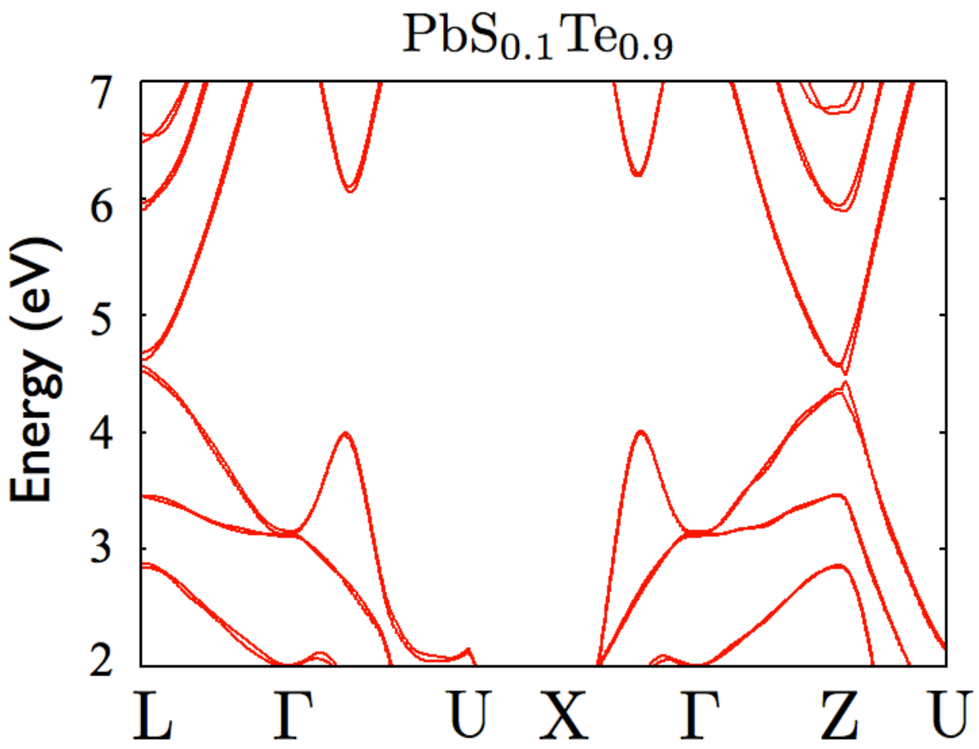

FIG. 4: VCA bandstructure for x=0.10

It is important to note that within the Virtual Crystal Approximation (VCA) the bandstructures are rather different from those within CPA. For instance, in Fig. 4 we report the bands for $PbS_{0.1}Te_{0.9}$, where a Rashba spin-splitting around the Z point is clearly evident, at variance with what happens within CPA, as a consequence of the disorder self-energy. The same discrepancy holds for x=0.18 and x=0.30.

[1] K. Kobayashi, T. Ohtsuki, K.-I. Imura and I. F. Herbut, Phys. Rev. Lett. **112**, 016402 (2014)

[2] Y. Ominato and M. Koshino, Phys. Rev. B. **89**, 054202 (2014)

[3] Z. Wang and B. A. Bunker, Phys. Rev. B 46, 11277 (1992)

[4] A. I. Lebedev and I. A. Sluchinskaya, Ferroelectrics 157, 275 (1994)

[5] W. D. Wilson, R. D. Hatcher, G. J. Dienes, and R. Smoluchowski, Phys. Rev. B 161, 888 (1967)

[6] J. J. van der Klink and S. N. Khanna, Phys. Rev. B 29, 2415 (1984)
